# Differentiated expression of long non-coding RNA-small nucleolar RNA host gene 8 in atherosclerosis and its molecular mechanism

**DOI:** 10.1080/21655979.2021.1979441

**Published:** 2021-09-24

**Authors:** Shuang Wang, Jianchao Li, Aimei Chen, He Song

**Affiliations:** a Department of Emergency Neurology, Yidu Central Hospital of Weifang, Weifang, Shandong China; b Department of Traditional Chinese Medicine, Yidu Central Hospital of Weifang, Weifang, Shandong China; c Department of Emergency, Yidu Central Hospital of Weifang, Weifang, Shandong China

**Keywords:** Atherosclerosis, lncSNHG8, miR-224-3p, vascular smooth muscle cell

## Abstract

Atherosclerosis (AS) is one of the most common cardiovascular diseases, and the incidence is increasing year by year. Many studies have shown that long non-coding RNA plays a vital role in the pathogenesis of AS. This study aimed to explore the role and mechanism of lncRNA-small nucleolar RNA host gene 8 (SNHG8) in AS. The expressions of serum lncSNHG8 and miR-224-3p were determined by quantitative real-time polymerase chain reaction (qRT-PCR). The diagnostic meaning of lncSNHG8 in AS was estimated by Receiver operating characteristic (ROC) curve. The correlation between lncSNHG8 and various clinical indicators, as well as miR-244-3p was evaluated by Pearson correlation coefficient analysis. Cell proliferation and migration were estimated by cell counting kit-8 (CCK-8) and Transwell assay. The interaction between lncSNHG8 and miR-224-3p was proved by luciferase reporter gene assay. The expression level of lncSNHG8 was increased in AS patients, while miR-224-3p expression was decreased. The ROC curve indicated that lncSNHG8 with high serum expression had the ability to distinguish AS. Pearson correlation coefficient exhibited that the level of miR-224-3p was negatively correlated with the level of lncSNHG8. The results of cell experiments indicated that inhibition of the expression of lncSNHG8 significantly inhibited the proliferation and migration of vascular smooth muscle cells (VSMCs). Luciferase reporter gene experiments confirmed that there was a target relationship between lncSNHG8 and miR-224-3p. In conclusion, lncSNHG8 had high diagnostic value for AS. It promoted the proliferation and migration of VSMCs by adsorption and inhibition of miR-224-3p.

## Introduction

The worldwide impact of cardiovascular diseases is far greater than other diseases, and the cost of treatment on cardiovascular diseases has placed a heavy economic burden on society and families [1]. Atherosclerosis (AS) is the main pathological change of cardiovascular disease, which is considered as the root cause of cardiovascular disease and has become an important factor affecting human health [2,3]. The development of AS involved changes and interactions of cell components in the vascular wall, such as dysfunction of vascular endothelial cells [4], phenotypic transformation and dysfunction of vascular smooth muscle cells (VSMCs) [5], and activation of macrophages [6]. Looking for the potential biomarkers in the complicated pathological changes of AS is crucial for the prevention and treatment of the occurrence and development of AS.

With the rapid development of gene sequencing technology, more and more long non-coding RNAs (lncRNAs) have been discovered, and their diverse biological functions are gradually being recognized by everyone [7]. Plenty of evidence illustrated that lncRNA participated in the growth, development, differentiation, and apoptosis of organisms through various mechanisms, and thereby playing a crucial role in the occurrence of diseases [8]. In AS, abnormal proliferation of VSMCs is a key step in the development of AS. Wu et al. found that lncRNA-p21 played an anti-AS role by inhibiting the proliferation of VSMCs in vitro [9]. Dysfunction of endothelial cells was considered as an important cause of AS, and nitric oxide synthase (eNOS) is a sign of normal endothelial cell function [10]. It was reported by Miao et al. that lncRNA-LEENE enhanced the anti-inflammatory ability of endothelial cells by improving the expression of eNOS mRNA and protein, thus producing an anti-AS effect [11]. These previous findings demonstrated the complex coordination role of lncRNA in AS. Intriguingly, lncSNHG8 (small nucleolar RNA host gene 8) was found to be associated with cardiovascular disease. In a recent study by Zhuo et al., they revealed that lncSNHG8 may be involved in acute myocardial infarction (AMI) through the regulation of SOCS3 expression by sponging miR-411-5p [12]. Zhang et al. reported that lncSNHG8 promoted myocardial cell injury and inflammatory response by regulating the NF-kB signaling pathway and participated in the regulation of myocardial infarction (MI) [13]. However, it is unclear whether and how lncSNHG8 participates in AS, which requires further study and analysis.

According to the evidence from previous studies, we concluded that SNHG8 may play a regulatory role in AS. To explore the expression of SNHG8 in AS patients, this study analyzed the serum level of SNHG8 in AS patients and further evaluated the diagnostic value of SNHG8 for AS. Besides, our study evaluated the effect of SNHG8 on the proliferation and migration of HA-VSMCs and further investigated its possible mechanism. In a word, current studies suggest that abnormally expressed SNHG8 may have some clinical diagnostic value for AS and SNHG8 may regulate cell proliferation and migration by targeting miR-224-3p.

## Methods and materials

### Study population and sample collection

Eighty-three patients diagnosed with AS were selected as the AS patient group, and 84 cases without AS were selected as the control group. All research subjects and their families in this study were signed an informed consent form. This study has been approved by the ethics committee of Yidu Central Hospital of Weifang. The inclusion criteria for AS patients were determined according to the 2017 European Society of Cardiology Guidelines for the Diagnosis and Management of Peripheral Artery Disease: CIMT < 0.9 mm was presented as normal, 0.9 mm ≤ CIMT < 1.3 mm was presented as intima thickening, CIMT > 1.3 mm was presented as plaque formation [14]. Inclusion criteria for the control group were the people who had no cerebrovascular history and CIMT < 0.9 mm. Patients with a diagnosis of severe cardiovascular disease, such as hemangioma, acute myocardial infarction, heart failure, angina pectoris, stroke, were excluded from the study. Venous blood of all subjects was collected, anticoagulated and centrifuged, and the supernatant obtained was stored in a − 80°C refrigerator for later use the detection of biochemical indexes, such as SNHG8 and miR-224-3p expression. Basic clinical characteristics of the subjects were collected and recorded for further analysis.

### Cell culture and cell transfection

Human aortic vascular smooth muscle cell lines (HA-VSMCs) were purchased from Shanghai Institute of Biochemistry and Cell Biology (SIBCB, Shanghai, China) and were grown in DMEM containing 10% fetal bovine serum (FBS) and 1% antibiotics (streptomycin 100 μg/mL, plus penicillin 100 U/mL) in an incubator. GenePharma (Shanghai, China) was responsible for the synthesis of small interfering RNA negative control (si-NC) and small interfering RNA against SNHG8 (si-SNHG8), miR-224-3p mimic, mimic NC, miR-224-3p inhibitor and inhibitor NC. Cell transfection was achieved by Lipofectamine 2000 (Invitrogen, USA) in accordance with the manufacturer’s instructions.

### RNA extraction and qRT-PCR

The level of SNHG8 was determined by qRT-PCR, and detailed steps referred to previously published articles [15]. Trizol reagent was used for RNA extraction from serum and cells. The reverse transcription system was prepared in strict accordance with the instructions of SuperScript II Reverse Transcriptase kit and PrimeScript™ RT reagent Kit, and the extracted total RNA was reverse transcribed into cDNA under appropriate conditions. The qRT-PCR reaction system was prepared according to the instructions on the miScript SYBR® Green PCR kit. The concentration and purity of RNA were measured by Nano Drop 2000 C spectrophotometer. In addition, when the ratio of OD260/OD280 was between 1.7 and 2.2, the purity of RNA was proved to meet the requirements. RNA integrity was assessed using an RNA 6000 Nano kit, in which samples with a RIN (RNA integrity number) lower than 7 were excluded. GADPH and U6 were defined as internal references for lncSNHG8 and miR-224-3p, respectively. The relative gene expression was normalized according to 2^−ΔΔCt^ method.

### CCK-8 assay

CCK-8 assay was performed for the evaluation of cell proliferation [16]. Briefly, HA-VSMCs were incubated in 96-well plates for cell transfection, and cell proliferation was detected by cell counting kit-8 (CCK-8) assay at 0 h, 24 h, 48 h, and 72 h after transfection. The brief steps were as follows: at each preset time point, 10 μL of CCK-8 solution was added to each well and incubated in the dark for 2 hours. The OD value at 450 nm was then measured using a microplate reader.

### Transwell assay

Cell migration was estimated by Transwell assay as previously published [17]. The transfected HA-VSMCs in logarithmic growth phase were suspended in FBS-free medium, and the cell suspension was seeded into the upper chamber. The lower chamber was added with complete medium containing FBS. The prepared device was placed in a cell culture incubator for 24 hours, Cells migrated to the bottom of the inserts were fixed with methanol and stained with crystal violet for 20 min. Finally, the number of migrated cells were calculated in six random fields of the microscope.

### Luciferase reporter gene assay

The interaction between SNHG8 and miR-224-3p was verified by Luciferase Reporter gene assay [18]. Steps are as follows: The 3ʹ-UTR fragments of lncSNHG8 were cloned into pLG3 vector to synthesize wild-type (WT) and mutation-type (MUT) vectors. HA-VSMCs were seeded into 12-well plate and cultured for 24 h. Then, the WT or MUT reporter vectors were co-transfected with miR-224-3p mimic, miR-224-3p inhibitor, mimic NC, and inhibitor NC into HA-VSMCs by Lipofectamine 2000 for 48 h. HA-VSMCs were harvested, and the luciferase activity was detected by a dual luciferase reporting system (Promega, USA). Renilla luciferase activity was selected as control.

### Data analysis

Data analysis was performed by GraphPad Prism version 7.0 and SPSS 21.0. The differences in groups were analyzed by student t test and one-way ANOVA. ROC curve evaluated the diagnostic significance of lncSNHG8 in AS. The correlation analysis was completed using Pearson correlation coefficient. *P* < 0.05 represents a significant difference and the data was represented as mean ± standard deviation (SD). Each experiment was repeated in triplicate.

## Results

### Comparison of baseline data

A total of 167 subjects were included in this study, including 83 AS patients and 84 controls. Baseline data for all subjects are shown in the Table 1, there was no statistically significant difference in gender, age, BMI, TC, HDL, LDL, TG, and FBG (*P* > 0.05). In addition, we can clearly notice that SBP, DBP, cfPWV, and CIMT in AS group were higher than those in the control group (*P* < 0.001).

**Table 1.** Basic clinical information of the subjects

Indicators	Control (n = 84)	Atherosclerosis (n = 83)	*P* value
Gender (males/females)	47/37	46/37	0.945
Age (years)	53.80 (6.57)	53.75 (6.17)	0.959
BMI (kg m^−2^)	25.08 (2.07)	25.46 (2.26)	0.256
TC (mg dl^−1^)	195.85 (16.93)	201.29 (30.26)	0.153
HDL (mg dl^−1^)	50.50 (5.28)	49.23 (8.31)	0.241
LDL (mg dl^−1^)	120.33 (15.21)	124.45 (18.98)	0.123
TG(mg dl^−1^)	145.41 (19.79)	151.73 (29.08)	0.102
FBG(mmol l^−1^)	5.45 (0.96)	5.47 (1.17)	0.917
SBP(mm Hg)	129.70 (18.22)	152.87 (20.31)	<0.001 (0.0000)
DBP(mm Hg)	83.50 (9.96)	89.17 (9.64)	<0.001 (0.0002)
cfPWV (m s^−1^)	6.54 (1.70)	12.50 (3.52)	<0.001 (0.0000)
CIMT (mm)	0.60 (0.15)	1.23 (0.14)	<0.001 (0.0000)

Abbreviations: BMI, body mass index; TC, total cholesterol; HDL, high-density lipoprotein; LDL, low density lipoprotein; TG, triglyceride; FBG, fasting blood-glucose; SBP, systolic blood pressure; DBP, diastolic blood pressure; cfPWV, carotid-femoral pulse wave velocity; CIMT, carotid intima-media thickness. Data are expressed as n or mean (standard deviation).

### Serum level of lncSNHG8 in AS patients

**Figure 1.**
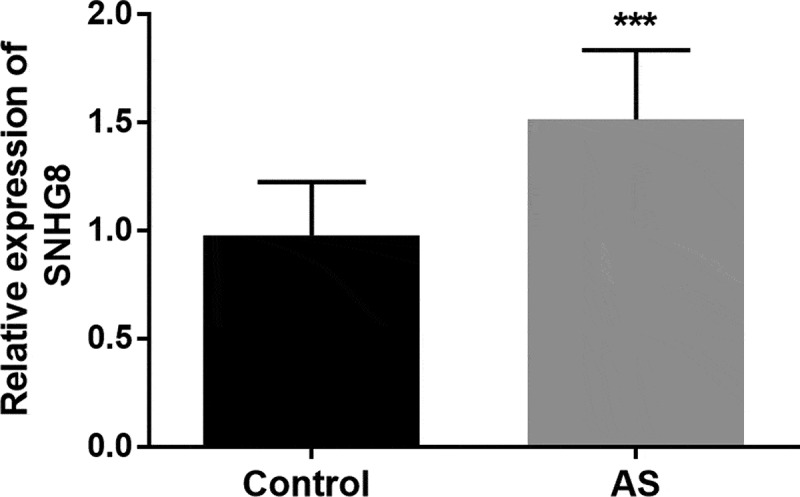
LncSNHG8 expression was increased in AS patients. ***P < 0.001

To verify whether the level of SNHG8 in the serum of subjects is abnormal or not, the level of lncSNHG8 in all subjects was measured by qRT-PCR. As shown in **Figure 1**, a significant increase in expression levels was observed in the AS group in comparison to control group (*P* < 0.001).

### Diagnostic value and correlation analysis

**Figure 2.**
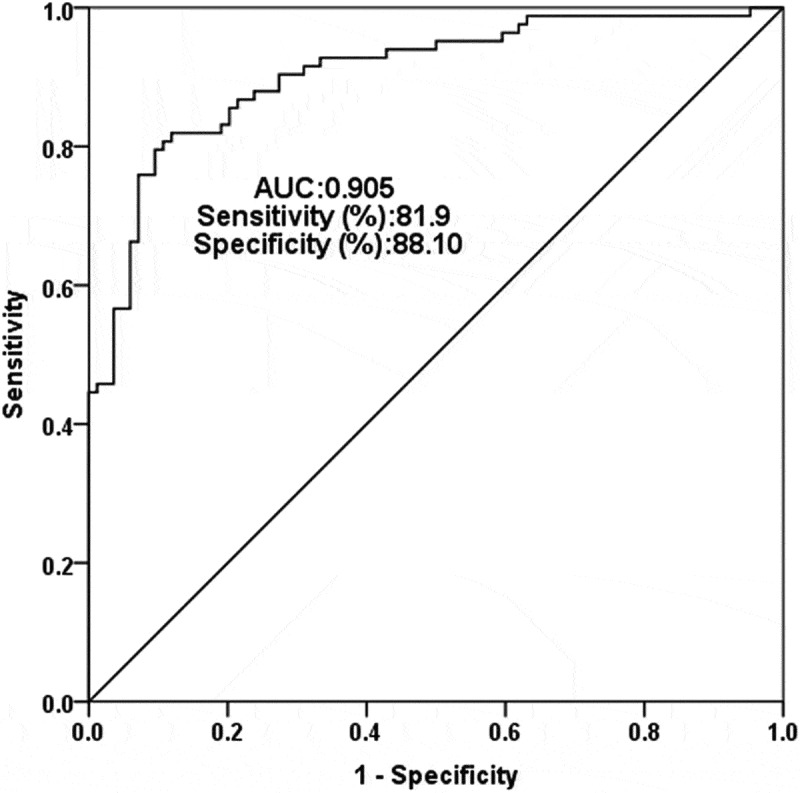
Receiver operating characteristic curve analysis of the AUC, specificity, and sensitivity of lncSNHG8 in AS

In view of the abnormal expression of SNHG8 in the serum of AS patients, ROC curve analysis was performed to evaluate the clinical diagnostic value of SNHG8 for AS. The area under curve (AUC) of the ROC curve is closer to 1, indicating higher diagnostic accuracy and diagnostic value. The results showed that the AUC was 0.905, the sensitivity was 81.9% and the specificity was 88.1% (**Figure 2**), indicated that lncSNHG8 has a high recognition ability and diagnostic significance for AS. Pearson correlation coefficient method was used to survey the correlation in lncSNHG8 and clinical indicators in AS patients. In Table 2, there was: a positive and strong correlation between lncSNHG8 and cfPWV (r = 0.792, *P* < 0.001), a positive and strong correlation between lncSNHG8 and CIMT (r = 0.720, *P* < 0.001), a positive and moderate correlation between lncSNHG8 and DBP (r = 0.515, *P* < 0.001), a positive and weak correlation between lncSNHG8 and SBP (r = 0.321, *P* = 0.003).

**Table 2.** Correlation between lncRNA SNHG8 and clinical characteristics

Characteristics	Correlation with lncRNA SNHG8 (r)	*P-value*
TC (mg dl^−1^)	0.132	0.235
HDL (mg dl^−1^)	−0.052	0.642
LDL (mg dl^−1^)	0.168	0.129
TG(mg dl^−1^)	0.159	0.152
FBG(mmol l^−1^)	0.032	0.776
SBP(mm Hg)	0.321	0.003
DBP(mm Hg)	0.515	<0.001
cfPWV (m s^−1^)	0.792	<0.001
CIMT (mm)	0.720	<0.001

Abbreviations: TC, total cholesterol; HDL, high-density lipoprotein; LDL, low density lipoprotein; TG, triglyceride; FBG, fasting blood-glucose; SBP, systolic blood pressure; DBP, diastolic blood pressure; cfPWV, carotid-femoral pulse wave velocity; CIMT, carotid intima-media thickness.

### Effects of lncSNHG8 knockdown on HA-VSMCs cell function

**Figure 3.**
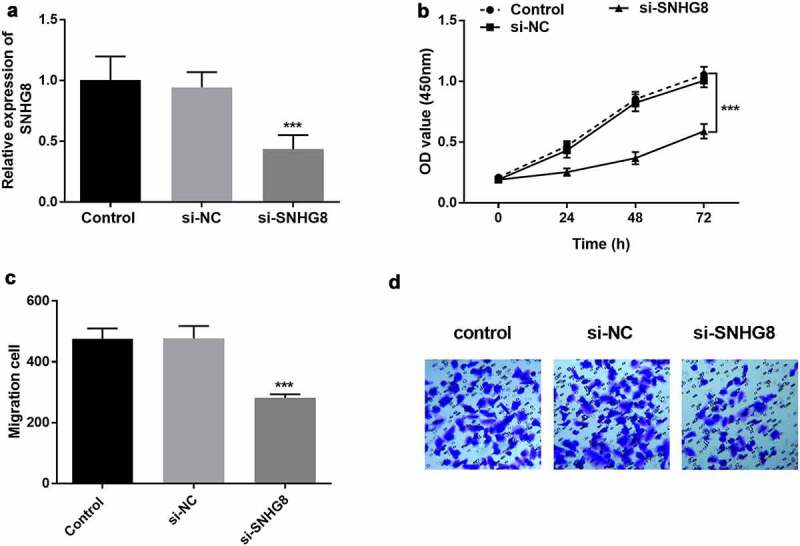
(A) PCR analysis of down-regulation efficacy after transfection with si-SNHG8 in HA-VSMCs. (B) CCK-8 analysis of decreased cell viability in si-SNHG8 group. (C) Transwell analysis of reduced cell migration after transfected with si-SNHG8. (D) Transwell image of cell migration after transfected with si-SNHG8. ***P < 0.001

The effect of SNHG8 on the proliferation and migration of HA-VSMCs was evaluated by in vitro cell transfection. According to the clinical results, the expression of lncSNHG8 in AS patients was increased compared with control group, and it was preliminarily judged that the high expression of lncSNHG8 had a promoting effect on AS. Therefore, *in vitro* cell experiments were conducted to verify the effect of down-regulation of lncSNHG8 on cell biological functions. It could be seen that the expression of lncSNHG8 in HA-VSMCs was effectively suppressed after transfected with si-SNHG8 (**Figure 3A**, *P* < 0.001). CCK-8 assay illustrated that cell growth was significantly inhibited by lncSNHG8 knockout (**Figure 3B**, *P* < 0.001). Transwell assay exhibited that cell migration was markedly decreased after down-regulation of lncSNHG8 (**Figure 3**** C-D**, *P* < 0.001).

### LncSNHG8 directly sponges with miR-224-3p in HA-VSMCs

**Figure 4.**
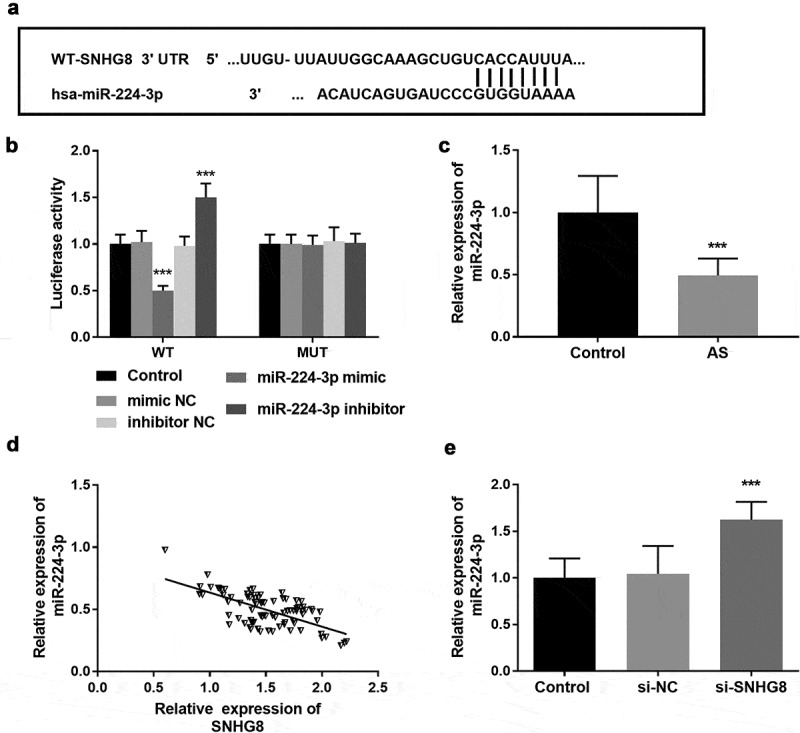
(A) The binding site of lncSNHG8 and miR-224-3p. (B) Luciferase activity analysis after co-transfected with WT or MUT vector and miR-224-3p mimic or inhibitor. (C) Serum miR-224-3p expression was decreased in AS patients. (D) Correlation analysis manifested that miR-224-3p was negatively correlated with lncSNHG8. (E) PCR analysis of overexpression efficacy of miR-224-3p after transfection with si-SNHG8 in HA-VSMCs. ***P < 0.001

To explore the potential mechanism of SNHG8 regulating cell function, bioinformatics software was used to predict the target genes of SNHG8. In HA-VSMCs, the targeting relationship between SNHG8 and miR-224-3p was verified by luciferase reporter gene. Possible downstream molecules of lncSNHG8 were predicted by through Starbase V2.0, and the obtained data showed that there was a binding site between miR-224-3p and lncSNHG8, which was shown in **Figure 4A**. This result was confirmed by luciferase reporter gene assay. In WT group, miR-224-3p mimic inhibited luciferase activity, but this phenomenon was not observed in the MUT group (**Figure 4B**, *P* < 0.001). Meanwhile, the expression of miR-224-3p in serum samples of AS patients was detected, and the results showed that the level of miR-224-3p was decreased in AS patients compared with the control group (**Figure 4C**, *P* < 0.001), and it was found that the expression of miR-224-3p was negatively correlated with lncSNHG8 (**Figure 4D**, *P* < 0.001). Next, we also verified the relationship between miR-224-3p and lncSNHG8 in HA-VSMCs. The PCR results demonstrated that inhibition of lncSNHG8 could correspondingly increase the expression of miR-224-3p (**Figure 4E**, *P* < 0.001). The above data together indicated that there was an association between lncSNHG8 and miR-224-3p and that lncSNHG8 was a sponge of miR-224-3p in HA-VSMCs.

## Discussion

AS is a complex chronic angio-inflammatory lesion caused by a variety of factors and is the basis of cardiovascular and cerebrovascular diseases such as myocardial infarction, stroke, unstable angina pectoris and sudden cardiac death, which has brought a great threat to human health [19]. LncRNA regulates AS by controlling transcription, activating enhancers, acting as microRNA sponges, binding proteins, and protein translation [20]. In this study, it was found that lncSNHG8 presented a trend of high expression in the serum of AS patients, while the expression of miR-224-3p was significantly decreased. The high level of lncSNHG8 was positively correlated with blood pressure and other indicators, and negatively correlated with the level of miR-224-3p. And *in vitro* experiment results revealed that lncSNHG8 may play a role in regulating cell function in HA-VSMCs through sponging with miR-224-3p and the knockdown of lncSNHG8 effectively inhibited cell proliferation and migration.

LncRNA was once considered to be a regulator of cardiovascular disease progression. Several studies reported that lncANRIL [21] and lncGAS5 [22] regulated AS-related biological processes, including lipid deposition and endothelial homeostasis. LncSNHGs are a stable cytoplasmic lncRNAs with large family members, among which, lncSNHG8 has been identified to be abnormally expressed in a variety of cancers [23]. For example, Xu et al. found that lncSNHG8, as an oncogene of breast cancer, accelerated the proliferation, migration and invasion of breast cancer cells [24]. Dong et al. reported that lncSNHG8 promoted the spread of liver cancer and lung metastasis through sponged with miR-149 [25]. Several previous studies have confirmed that lncSNHG8 was abnormally expressed in MI, and unstable atherosclerotic plaques are the direct cause of MI [12,13]. In the current study, it was found that the relative expression of lncSNHG8 was upregulated in AS samples. This result was consistent with that of Zhang et al. in serum of MI patients. Subsequent data demonstrated that the level of lncSNHG8 was positively correlated with DBP, SBP, cfPWV and CIMT. All these results proved that lncSNHG8 was indeed connected with AS.

Studies have shown that VSMCs can be transformed from contractile phenotype to synthetic phenotype in atherosclerotic lesions, showing high proliferation and migration rate, which plays an important role in cardiovascular diseases [26]. VSMCs are the main cellular components of atherosclerotic plaques, and abnormal proliferation of VSMCs is a key step in plaque formation [27]. In this study, the biological functions of lncSNHG8 on cells were estimated using HA-VSMCs via cell transfection. It could be seen that the knockdown of lncSNHG8 suppressed HA-VSMCs proliferation and migration, and this result also reflected the promotion effect of SNHG8 overexpression in AS from the side. In terms of mechanism, lncRNAs regulated gene expression through the sponging effect of miRNA. The role of miRNA in cardiovascular diseases has always been a research hotspot. Woo et al. found 13 miRNAs related to VSMCs in aortic wall tissues of patients undergoing coronary artery bypass grafting. They also found that overexpression of miR-30b-5p affected the proliferation of VSMCs and may be involved in their differentiation [28]. Similarly, this study showed that miR-224-3p had the binding site with lncSNHG8. While miR-244 was first identified as a tumor suppressor, Miller et al. found that miR-224 was significantly under-expressed in atherosclerotic plaques in patients with coronary atherosclerosis [29]. Xu et al. demonstrated down-regulation of miR-224 in a rat aggravated atherosclerotic plaque formation and vascular remodeling in acute coronary syndromes by activating the TGF-β/Smad pathway [30]. Moreover, Wang et al. reported that in in vivo experiments, it was observed that HDAC1 reduced the accumulation of ROS and apoptosis of endothelial cells in AS animal models by upregulation of miR-224-3p, thus inhibiting the process of AS [31]. In the AS samples of our study, miR-224-3p expression was detected to be down-regulated, and previous studies mentioned above also support our experimental results. In addition, the expression of miR-224-3p was negatively correlated with lncSNHG8. And luciferase reporter gene experiments confirmed that the binding of miR-224-3p mimic with WT-SNHG8-3ʹ UTR in HA-VSMCs significantly unbraced the luciferase activity, and it had no effect on MUT group. These above results revealed that lncSNHG8 might act as a sponge of miR-224-3p to provide HA-VSMCs with a role in promoting cell viability and migration.

It should be noted that the *in vitro* cell experiments in this study only preliminarily verified the effect of SNHG8 on VSMCs *in vitro*. Although our initial purpose was to verify our findings in clinical research through cell experiments, and we have achieved the expected results. But in fact, the data provided by actual clinical samples may not be as relevant as the results of *in vitro* studies. Like Carvalho et al.’s study, they found significantly elevated levels of 5 of 12 characteristic ceramides in plasma of patients with AMI, but no such trend was found in aortic biopsy. In addition, the concentration of 12 ceramides increased in the rat model of myocardial infarction [32]. We believe that the organism is an extremely complex environment, and under the joint control of many factors, the results of *in vitro* experiments may not be well presented in *in vivo* study. In addition, the treatment of AS patients often requires oral antiplatelet drugs or other anticoagulants, which have been proved to affect the expression of miRNA. Therefore, for this study, we still need to design more experiments to verify and support our findings.

Several limitations of the current study cannot be ignored. Firstly, we only explored the effect of lncSNHG8 on the function of HA-VSMCs, we did not investigate the effect of miR-224-3p on cell function. According to our inference of the results, it was inferred that miR-224-3p has an impact on the function of HA-VSMCs, and it needs to be further verified. Secondly, the ceRNA was not complete. This study revealed the interaction between lncSNHG8 and miR-224-3p, but we did not continue to explore the expression of the target genes associated with miR-224-3p. Thirdly, the clinical diagnostic performance of SNHG8 still needs to be further evaluated by expanding the sample size to exclude possible deviations caused by the small sample size and the recruitment of subjects from a single clinical center.

## Conclusion

In summary, our results showed that lncSNHG8 promoted the proliferation and migration of HA-VSMCs by sponging miR-224-3p, and the highly expressed lncSNHG8 had more accurate diagnostic significance for AS, which may provide a new target for the intervention and treatment of AS in the future.

## Highlights

1. The level of serum SNHG8 in AS patients was increased, while the level of miR-224-3p was decreased.

2. SNHG8 has certain clinical diagnostic value for AS.

3. Down-regulation of SNHG8 inhibited cell proliferation and migration of HA-VSMCs.

4. SNHG8 negatively regulates miR-224-3p in HA-VSMCs.
